# Failed resuscitation of a newborn due to congenital tracheal agenesis: a case report

**DOI:** 10.4076/1757-1626-2-7212

**Published:** 2009-07-17

**Authors:** Koen P Dijkman, Peter Andriessen, Gesina van Lijnschoten, Feico J Halbertsma

**Affiliations:** 1 Department of Neonatology, Máxima Medical CenterPO Box 7777, 5500 MB, VeldhovenThe Netherlands; 2 Pathology Laboratory [Stichting Laboratoria voor Pathologie en Medische Microbiologie (Stichting PAMM)]Michelangelolaan 2, 5623 EJ, EindhovenThe Netherlands

## Abstract

Tracheal agenesis is a rare congenital condition. It usually presents as an unexpected emergency during resuscitation of a newborn in the delivery room. The condition is almost always fatal in the resuscitation phase, but also when the neonate survives the long term prognosis remains poor. We present a case of tracheal agenesis, discuss its presenting symptoms and possibilities for antenatal diagnosis and review the therapeutic options.

## Introduction

**Figure 1.**
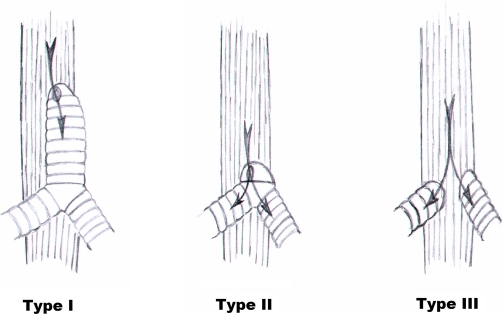
Floyd’s classification of tracheal agenesis.

Tracheal agenesis is a very rare condition, with an incidence of approximately 1 in 50.000 [1]. It was first described in the literature in 1900 by Payne [2]. A classification of various phenotypes of tracheal agenesis was proposed by Floyd in 1962 [3]: Type I: atresia of the proximal trachea, with a distal segment of the trachea and normal main bronchi and a tracheo-oesophageal fistula; Type II: atresia of the complete trachea, normal carina and main bronchi with or without tracheo-oesophageal fistula; Type III: the main bronchi arise independent from the oesophagus (Figure 1). Type I and Type III each account for 20% of the cases of tracheal agenesis, Type II accounts for 60%. Despite the introduction of a more extended classification by Faro in 1979 distinguishing 7 phenotypes [4], Floyd’s original classification remains most commonly used. We describe here a case of Floyd’s type II tracheal agenesis. We discuss its antenatal presenting symptoms, possibilities for antenatal diagnosis and therapeutic options.

## Case presentation

**Figure 2.**
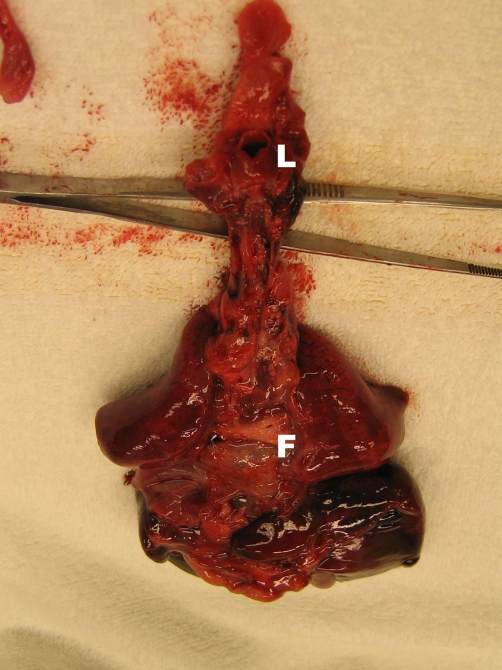
Dorsal view of the mediastinum after removal of the oesophagus, showing the normal larynx (**L**) and the bronchi with the fistula (**F**).

**Figure 3.**
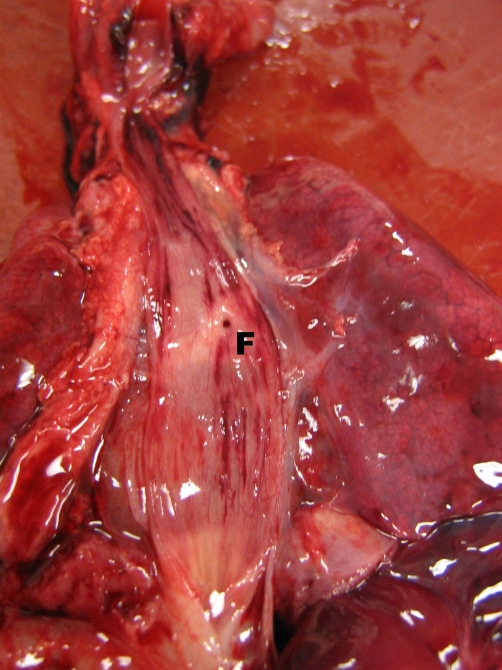
Dorsal view of the mediastinum after opening the oesophagus, showing the tracheo-oesophageal fistula (**F**).

A 27-year old Dutch Caucasian primigravida was referred to our hospital at 30 3/7 weeks gestation because of preterm premature rupture of the membranes. There was a history of polyhydramnion. Fetal ultrasonography had revealed no abnormalities such as abnormal lung development or absent gastric filling. A course of antenatal steroids was given to induce lung maturation. At 31 weeks gestation delivery was imminent and a boy was born. Despite the fact that there were no signs of fetal distress during labour the newborn baby had a poor start: there were signs of severe respiratory distress and there was no audible cry. Positive pressure ventilation with a mask was initiated, which not resulted in thoracic excursions and lead to further deterioration of the newborn. Apgar score was 1 after 1 minute and tracheal intubation was attempted with an endotracheal tube size 3.0. Despite good visualisation of the epiglottis and vocal cords, the tube could not pass any further than the vocal cords. Subsequent attempts with a tube 2.5 and tube 2.0 failed in a similar way. While breathing spontaneous with CPAP and 100% oxygen there were periods of visible chest excursions and brief improvement of the condition of the patient. Flexible tracheoscopy showed a blind ending larynx. Based on this finding combined with profound hypoxia and bradycardia resuscitation attempts were ceased. Post mortem analysis showed a normal larynx, complete absence of the trachea, a normal developed carina and main bronchi and a small fistula (diameter 1 mm) between the distal oesophagus to the right main bronchus (Figures 2 and 3). Thus classifying this case as a type II tracheal agenesis according to Floyd. Furthermore, the post mortem analysis revealed a lobation defect of the right lung. The right lung consisted of one singular lobe. Post mortem radiology studies revealed a hemivertebra of the 11^th^ thoracic vertebra. No further congenital abnormalities were found.

## Discussion

This case illustrates a typical example of presentation and clinical course of tracheal agenesis. Although this is a rare condition to this date over a 100 cases have been reported in the literature. Tracheal agenesis can present as a single organ malformation, but in 93% more congenital abnormalities are encountered, including lung lobation and vertebral defects as observed in this patient [5].

The embryogenesis of congenital tracheal abnormalities is still incompletely understood. In the 3^rd^ week of embryogenesis the fetal trachea and oesophagus develop as the foregut reduces in size. Two cranial and one caudal fold appear in the primitive foregut. Caudal movement of the cranial folds and cranial movement of the tracheo-oesophageal fold reduces the size of the foregut. After separation of the foregut, a significant elongation of the trachea and oesophagus follows. In tracheal agenesis the developmental movements of the folds are disturbed. Displacement of the tracheo-oesophageal space in dorsal direction results in differentiation in the oesophagus [6].

As the overall outcome of tracheal agenesis is poor, antenatal diagnosis of the condition is very important. This allows the physicians to inform the future parents, assess the situation and consider treatment strategies. Antenatal identification of tracheal agenesis remains difficult. The antenatal presenting symptom is often that of a polyhydramnion, probably due to functional oesophagus obstruction resulting from abnormal laryngeal anatomy. Unfortunately it is not possible to make the diagnosis by antenatal ultrasonography. Ultrasonography however can give certain clues toward a diagnosis. In complete obstruction of the trachea uniform hyperechogenic lungs may be seen with flattening of the diaphragm due to over distension of the obstructed lungs [7]. Furthermore ultrasonography can reveal other anomalies that are associated with tracheal agenesis. The diagnosis can be made by magnetic resonance imaging [8]. While MRI will confirm the diagnosis in selected cases, where abnormalities found on ultrasonography will warrant further investigation, it will be difficult to justify MRI in all cases of isolated polyhydramnion to rule out the possibility of tracheal agenesis.

The clinical picture is usually that of a newborn with immediate respiratory distress at birth and no audible cry. Direct laryngoscopy reveals a normal larynx but there is an impossibility to advance an endotracheal tube beyond the larynx. Temporary successful ventilation over a tube placed in the oesophagus may be possible in the presence of a fistula.

A systematic surgical approach does not exist but limited success of surgical management is reported [9]. Emergency management in the presence of a tracheo-oesophageal fistula or a Floyd type III tracheal agenesis may consist of temporarily using the oesophagus as an airway, and separating the digestive tract and the respiratory tract by oesophageal banding. A gastrostomy can be used for enteral feeding of these neonates. Prolonged ventilation through the oesophagus is not possible. Therefore, a solution has to be found in a form of tracheostomy, its success depending on the length of the distal trachea. In most cases long-term solutions for tracheal agenesis remain very limited and the outcome is almost always fatal [7,9,10]. It has been suggested that the use of tissue-engineered cartilage may improve the outcome, especially for the neonate with no or minor other congenital abnormalities [7].

In conclusion: tracheal agenesis is a severe congenital abnormality with distinct clinical symptoms. When suspected the diagnosis can easily be made after birth. The possibilities for prenatal diagnosis and postnatal treatment are limited.

## Abbreviation

CPAP: Continuous positive airway pressure
